# Clinical Presentation and Mortality Among Hospitalized People Living With HIV at a Tertiary Care Institute in Mumbai, India

**DOI:** 10.7759/cureus.104685

**Published:** 2026-03-04

**Authors:** Shrikala Acharya, Vijay Kumar Karanjkar, Shwetangi Shinde, Prashant Deshpande, Sachin Dhande, Sagar Koli, Amol Palkar, Lokesh Krishna, Priya Patil, Maninder S Setia

**Affiliations:** 1 Community Medicine, Lokmanya Tilak Municipal Medical College and General Hospital, Mumbai, IND; 2 Public Health, Mumbai Districts AIDS Control Society, Mumbai, IND; 3 Public Health, International Training and Education Center for Health (I-TECH), New Delhi, IND; 4 Data Management, International Training and Education Center for Health (I-TECH), New Delhi, IND; 5 Community Medicine, Grant Government Medical College and Sir JJ Group of Hospitals, Mumbai, IND; 6 Internal Medicine, Grant Government Medical College and Sir JJ Group of Hospitals, Mumbai, IND; 7 Epidemiology, Mahatma Gandhi Mission (MGM) Institute of Health Sciences, Navi Mumbai, IND

**Keywords:** aids-defining illness, epidemiology, hospitalization, hospital mortality, in-patient setting

## Abstract

Introduction: Despite global advancements in antiretroviral treatment and the widespread adoption of universal antiretroviral therapy (ART) initiation, hospitalization among people living with HIV (PLHIV) infection remains a persistent concern in many settings. A substantial proportion of hospitalized patients present with low CD4 lymphocyte count, opportunistic infections, or complications that arise from treatment interruption or untreated HIV infections. With increasing age among this population, the presence of non-communicable diseases (NCDs) has also become more common, making clinical management more complex. Despite overall progress in HIV care, severe illness requiring hospitalization continues to be driven by opportunistic infections associated with immune suppression, especially tuberculosis. This study sought to assess the demographic and clinical characteristics, causes of hospitalization, and hospital mortality among PLHIV, with a specific comparison between ART-experienced and treatment-naïve patients admitted to a tertiary care institute in Mumbai, India.

Methods: This cross-sectional study reviewed hospital records of 325 PLHIV inpatients in a tertiary care institute from June 2022 to May 2024. Demographic characteristics, ART status and duration, CD4 lymphocyte count at time of admission, causes of hospitalization, and patient outcomes were extracted from medical records. Causes of hospitalization were categorized as acquired immunodeficiency syndrome (AIDS)-defining illness (ADI) or non-ADI. Appropriate descriptive and inferential statistical analyses were performed.

Results: A total of 325 hospitalized PLHIV were included; 187 (57.5%) were on ART and 138 (42.5%) were treatment naïve. The median age was 44 years (IQR: 19). ADIs accounted for 147 (45.2%) of hospitalizations, with tuberculosis being the predominant cause. Treatment-naïve patients were more likely to present with severe immunosuppression, with 73 (52.9%) having CD4 lymphocyte counts of less than 200 cells/µL compared to 41 (21.9%) among ART-experienced patients. ADI-related hospitalizations were significantly associated with younger age, male sex, unmarried status, lower CD4 lymphocyte counts, and shorter duration on ART. Non-ADI hospitalizations were more common among older patients and those on ART for longer durations. Hospital mortality was seen in 28 (8.6%), predominantly among people with advanced HIV disease and ADIs, with tuberculosis being the most frequent opportunistic infection.

Conclusion: Despite widespread ART availability, ADIs, particularly tuberculosis, remain major drivers of hospitalization and hospital mortality among PLHIV in this setting. Simultaneously, the growing burden of non-ADIs and NCDs among ageing PLHIV highlights an ongoing epidemiological transition and the need for integrated, age-responsive HIV care models.

## Introduction

Advances in antiretroviral treatment in the past two decades have led to a substantial decline in new HIV infections and AIDS-related morbidity and mortality, transforming HIV from a fatal condition into a manageable chronic disease for people living with HIV (PLHIV) [1,2]. Despite this declining trend, hospitalization and hospital mortality among people with advanced HIV infections remain significant concerns, particularly in developing countries [3,4].

Evidence from multiple settings indicates that hospitalizations among PLHIV in developing countries remain largely driven by AIDS-defining illnesses (ADIs), most notably tuberculosis and other opportunistic infections, alongside severe bacterial infections [3,5,6]. At the same time, as PLHIV survive longer due to increased coverage and universal adoption of antiretroviral therapy (ART), there has been a growing contribution of non-AIDS-defining conditions to hospitalization, including cardiovascular, metabolic, renal, hepatic, and malignant diseases, especially in the developed countries [7,8]. This epidemiological transition reflects the combined effects of aging, chronic immune activation, long-term ART exposure, and traditional risk factors for non-communicable diseases (NCDs). Consequently, hospitalized PLHIV now represent a heterogeneous population with complex and overlapping infectious and non-infectious morbidities [3,9].

India contributes the second-largest burden of HIV infections worldwide, with an estimated 2.6 million PLHIV infection and 32,000 deaths in 2024 [10]. Over the past decade, the National AIDS Control Program has expanded ART access and adopted a policy of Test and Treat [11]. While these strategies are expected to reduce AIDS-related hospitalizations, several contextual factors may continue to influence inpatient morbidity patterns [12,13]. These include delayed diagnosis, interruptions in ART, suboptimal adherence, and service delivery challenges, such as those experienced during the COVID-19 pandemic [14,15]. As a result, a substantial proportion of hospitalized PLHIV may still present with advanced immunosuppression, while others are increasingly admitted for non-AIDS-related conditions associated with aging and NCDs [16,17].

Despite the scale of the HIV epidemic in India, systematic evidence on the demographic and clinical profiles and causes of hospitalization among PLHIV remains limited, particularly from tertiary health care settings in urban areas. If we have to achieve the goal of reducing the AIDS-related deaths by 90% from the 2010 baseline, as emphasized by the UNAIDS Global AIDS Strategy 2021-2026 [18], it is critical to collect this evidence for improving case management, optimizing ART program delivery, and strengthening linkages across levels of care. This study was undertaken to describe the demographic and clinical characteristics and causes of hospitalization among PLHIV, with a specific comparison between ART-experienced and treatment-naïve patients admitted to a tertiary care institute in urban India.

## Materials and methods

Study design

A cross-sectional descriptive study design was adopted for the analysis of morbidity patterns among PLHIV using existing medical records, making it appropriate for identifying hospitalization-related factors.

Study setting and timeline

This hospital-based cross-sectional study was conducted at a tertiary healthcare institute in Mumbai, India. The institute caters to a large urban and peri-urban population, serving as a regional centre of excellence for adult PLHIV under the Mumbai Districts AIDS Control Society (MDACS). The study included all inpatients admitted to the hospital who were known or newly diagnosed PLHIV. The study was conducted over two years, from June 2022 to May 2024. Preliminary activities included ethical approval and preparation of data extraction forms. Data collection was performed from hospital medical records, followed by data entry, cleaning, and statistical analysis. For PLHIV already on treatment, their treatment records about the date of ART initiation and CD4 count at the time of hospital admission were extracted from the Master line list kept at MDACS.

Study population and sampling

The reference population comprised all PLHIV seeking inpatient care in the tertiary care institute during the study period. The study population consisted of all adult PLHIV (≥18 years) admitted to any inpatient ward between June 2022 and May 2024. The inclusion of consecutive eligible cases ensured that the study population was a representative subset of PLHIV managed in similar tertiary settings. A total of 325 inpatient records meeting eligibility criteria were included through complete enumeration. No specific exclusion criteria were applied. Repeat hospitalizations were considered as separate admissions only if they occurred during different clinical episodes and were documented as distinct hospital events.

Variables and measurement

Data were extracted using a pre-designed structured proforma. The following variables were recorded: demographic variables (age at admission, gender, and marital status), HIV-related variables (ART initiation status, duration since ART initiation, and CD4 lymphocyte count at the time of hospitalization), and clinical variables (causes of hospitalization, presence of comorbidities, and outcome at discharge). Treatment status was classified as ‘treatment-naïve’ or ‘ART-experienced’. Individuals who were not receiving ART at the time of admission (n = 12), as well as those who had initiated ART within the preceding six months (n = 126), were grouped as treatment-naïve (total n = 138). This classification was based on prior evidence demonstrating that patients within the first six months of ART initiation exhibit morbidity patterns comparable to untreated individuals and are at a higher risk of hospitalization due to ADIs [5]. Causes of hospitalization were categorized into two groups based on WHO and National AIDS Control Organisation (NACO) criteria: ADIs (e.g., tuberculosis, Pneumocystis jirovecii pneumonia, cryptococcal meningitis, CNS toxoplasmosis, encephalopathy, oesophageal candidiasis, Cytomegalovirus retinitis, chronic diarrhea, and recurrent leishmaniasis), and non-ADIs. Missing data were observed for CD4 lymphocyte count in 33 patients (10.2%). A complete case analysis approach was used for comparisons involving CD4 categories. Potential confounding factors such as socioeconomic status, nutritional status, adherence to ART, time since HIV diagnosis, and prophylaxis for opportunistic infections could not be fully assessed due to limitations inherent to record-based data. Additionally, confirmatory diagnostic investigations for opportunistic infections were not available in all cases; hence, certain diagnoses were based on syndromic assessment, clinical signs and symptoms, and available laboratory or radiological findings.

Data collection instruments and techniques

Information was retrieved from hospital medical records, ART registers, and patient discharge summaries using a standardized data abstraction sheet. Confidentiality of all patient identifiers was strictly maintained by assigning unique study codes.

Data management and statistical analysis

Data were entered in Microsoft Excel 2019 (Redmond, WA, USA) and analyzed using R Version 4.5.2 (R Foundation for Statistical Computing, Vienna, Austria; https://cran.r-project.org/bin/windows/base/). Descriptive statistics such as frequencies and proportions were used for categorical data, and means and standard deviations were used to summarize linear data. The proportions of categorical variables were compared using the chi-square test or Fisher’s exact test for low expected cell counts, as applicable. A p-value <0.05 was considered statistically significant.

Ethical considerations

The study was conducted in accordance with the ethical standards of the Institutional Ethics Committee, Good Clinical Practice, and Declaration of Helsinki. All the participants provided a written informed consent prior to enrollment in the study. All data were stored in password-protected files accessible only to the investigators. The study was approved by the Mumbai Districts AIDS Control Society Ethics Committee (Ref No MDACS-IRB/6289).

## Results

A total of 325 PLHIV who had undergone hospitalization were included in the study, of whom 187 (57.5%) were ART experienced, and 138 (42.5%) were treatment naïve at the time of admission (Table 1). The median age was 44 years (IQR = 19). Among ART-experienced patients, the largest proportions were in the 31-45 years (68, 36.3%) and 46-60 years (71, 38%) age groups, while a similar pattern was observed among treatment-naïve patients (50, 36.2% and 52, 37.7%, respectively). Individuals aged >60 years constituted 19 (10.2%) of ART-experienced and 11 (8.0%) of treatment-naïve patients. Children and adolescents (<18 years) were uncommon in both groups. Gender distribution did not differ significantly by treatment status. Among ART-experienced patients, 106 (56.7%) were males, 79 (42.3%) were females, and two (1.1%) were transgender persons. Treatment-naïve patients were predominantly male (92, 66.7%), with females comprising 46 (33.3%). Marital status was similar across groups, with married individuals forming the majority (132, 70.6%, and 100, 72.5%, respectively) in both. Unmarried patients constituted 39 (20.9%) among ART-experienced and 29 (21.0%) among treatment-naïve patients. Widows or widowers accounted for 14 (7.5%) of ART-experienced patients and seven (5.1%) of treatment-naïve patients. A markedly significant difference was observed in the immunological status (p < 0.001) between the two groups. Among treatment-naïve patients, 73 (52.9%) had CD4 lymphocyte counts of less than 200 cells/µL, compared to 41 (21.9%) among ART-experienced patients. CD4 lymphocyte counts greater than 200 cells/µL were more frequent in the ART-experienced group (120, 64.2%) than among treatment-naïve patients (58, 42.0%).

**Table 1:** Demographics and clinical characteristics of patients (N = 325) *Fisher’s exact test

Characteristic	ART experienced (N = 1871)	Treatment naïve (N = 1381)	Total, n (%)	p-value*
Age group				
<18 years	3 (1.6%)	1 (0.7%)	4 (1.2%)	0.84
18-30 years	26 (13.9%)	24 (17.4%)	50 (15.4%)	
31-45 years	68 (36.3%)	50 (36.2%)	118 (36.3%)	
46-60 years	71 (38.0%)	52 (37.7%)	123 (37.9%)	
>60 years	19 (10.2%)	11 (8.0%)	30 (9.2%)	
Total	187 (57.5%)	138 (42.5%)	325 (100.0%)	
Gender				
Male	106 (56.7%)	92 (66.7%)	198 (60.9%)	0.08
Female	79 (42.3%)	46 (33.3%)	125 (33.3%)	
Transgender	2 (1.1%)	0 (0%)	2 (0%)	
Total	187 (57.5%)	138 (42.5%)	325 (100.0%)	
Marital Status				
Married	132 (70.6%)	100 (72.5%)	232 (71.4%)	0.83
Unmarried	39 (20.9%)	29 (21.0%)	68 (20.9%)	
Separated	2 (1.1%)	2 (1.5%)	4 (1.2%)	
Widow/Widower	14 (7.5%)	7 (5.1%)	21 (6.5%)	
Total	187 (57.5%)	138 (42.5%)	325 (100.0%)	
CD4 Category				
<200	41 (21.9%)	73 (52.9%)	114 (35.1%)	< 0.001
>200	120 (64.2%)	58 (42.0%)	178 (54.8%)	
Not available	26 (13.9%)	7 (5.1%)	33 (10.2%)	
Total	187 (57.5%)	138 (42.5%)	325 (100.0%)	

Table 2 shows the demographic and clinical characteristics of the patients by cause of hospitalization. Of the total cohort, 147 (45.2%) hospitalizations were due to ADIs and 178 (54.8%) were due to non-ADI causes. Age distribution differed significantly by ADI status (p = 0.02). Patients aged 18-30 years constituted a higher proportion of ADI hospitalizations (28, 19.0%) compared with non-ADI hospitalizations (22, 12.4%), whereas patients aged >60 years were more common among non-ADI hospitalizations (24, 13.5% vs. 6, 4.1%). The median age of patients admitted with ADIs was 42 years (IQR: 18), compared with 45 years (IQR: 16) among those admitted with non-ADIs, and this difference was statistically significant (p = 0.003). Gender was significantly associated with ADI status (p = 0.007). Males accounted for 102 (69.4%) of ADI hospitalizations, compared with 96 (53.9%) of non-ADI hospitalizations, while females comprised 44 (29.9%) of ADI hospitalizations and 81 (45.5%) of non-ADI hospitalizations. Marital status also differed significantly (p = 0.008). Unmarried patients formed a larger proportion of ADI hospitalizations (40, 27.3%) compared with non-ADI hospitalizations (28, 15.7%), whereas widows or widowers were more frequent among non-ADI hospitalizations (16, 9.0% vs. 5, 3.4%). The CD4 lymphocyte count category was significantly associated with ADI status (p < 0.001). Patients with CD4 counts greater than 500 cells/µL were more commonly admitted for non-ADI causes (66, 42.0%) than for ADI (20, 14.8%), while lower CD4 categories were relatively more represented among ADI hospitalizations. Duration on ART showed a strong association with ADI status (p < 0.001). ADI hospitalizations were more frequent among those on treatment for less than six months (77, 52.4%%) compared with non-ADI hospitalizations (61, 34.3%), while patients on ART for more than two years were more commonly admitted for non-ADI causes (109, 61.2% non-ADI vs. 50, 34.0% ADI).

**Table 2:** Demographic and clinical characteristics by cause of hospitalization (AIDS-defining vs non-AIDS-defining) *Fisher’s exact test

Cause of hospitalization	AIDS-defining illness (N = 1471)	Non-AIDS-defining illness (N = 1781)	Total, n (%)	p-value*
Age group				
<18 years	2 (1.4%)	2 (1.1%)	4 (1.2%)	0.02
18-30 years	28 (19%)	22 (12.4%)	50 (15.4%)	
30-45 years	56 (38.1%)	62 (34.8%)	118 (36.3%)	
45-60 years	55 (37.4%)	68 (38.2%)	123 (37.8%)	
>60 years	6 (4.1%)	24 (13.5%)	30 (9.2%)	
Total	147 (45.2%)	178 (54.8%)	325 (100.0%)	
Gender				
Male	102 (69.4%)	96 (53.9%)	198 (60.9%)	0.007
Female	44 (29.9%)	81 (45.5%)	125 (38.5%)	
Transgender	1 (0.7%)	1 (0.6%)	2 (0.6%)	
Total	147 (45.2%)	178 (54.8%)	325 (100.0%)	
Marital status				
Married	99 (67.3%)	133 (74.7%)	232 (71.4%)	0.008
Unmarried	40 (27.3%)	28 (15.7%)	68 (20.9%)	
Separated	3 (2.0%)	1 (0.6%)	4 (1.2%)	
Widow/Widower	5 (3.4%)	16 (9.0%)	21 (6.5%)	
Total	147 (45.2%)	178 (54.8%)	325 (100.0%)	
CD4 Category				
Under 200 cells/µL	76 (56.3%)	38 (24.2%)	114 (39.0%)	< 0.001
200–350 cells/µL	23 (17.0%)	23 (14.6%)	46 (15.8%)	
350–500 cells/µL	16 (11.9%)	30 (19.1%)	46 (15.8%)	
Over 500 cells/µL	20 (14.8%)	66 (42.0%)	86 (29.5%)	
Unknown	12	21	33	
Total	147 (45.2%)	178 (54.8%)	325 (100.0%)	
Duration on ART				
Under 6 months	77 (52.4%)	61 (34.3%)	138 (42.5%)	< 0.001
6 months to 2 years	20 (13.6%)	8 (4.5%)	28 (8.6%)	
More than 2 years	50 (34.0%)	109 (61.2%)	159 (48.8%)	
Total	147 (45.2%)	178 (54.8%)	325 (100.0%)	

Table 3 describes the distribution of clinical syndromes among patients grouped by CD4 lymphocyte count category at admission. ADIs were predominantly observed among patients with CD4 counts below 200 cells/µL. Tuberculosis constituted the most frequent ADI across all CD4 groups, with disseminated tuberculosis showing the strongest association with severe immunosuppression, as nearly two-thirds of such cases occurred among patients with CD4 counts below 200 cells/µL. Central nervous system and gastrointestinal opportunistic infections were also largely confined to patients with lower CD4 counts, while Pneumocystis jirovecii pneumonia and cytomegalovirus retinitis were more commonly observed among patients with CD4 counts below 200 cells/µL compared to those in higher CD4 categories. In contrast, non-ADIs were more frequent among patients with CD4 lymphocyte counts above 200 cells/µL. Medical conditions such as cardiovascular, hepatic, gastrointestinal, and renal disorders were predominantly observed in this group, with renal disorders showing the strongest skew towards higher CD4 counts. Surgical illnesses constituted a substantial proportion of non-AIDS-defining admissions and were most common among patients with CD4 lymphocyte counts above 200 cells/µL. Pregnancy-related hospitalizations and accidental injuries were also more frequently observed in patients with higher CD4 counts. Overall, our data demonstrates a gradient in which lower CD4 lymphocyte counts are associated with opportunistic infections and ADIs, whereas higher CD4 counts are associated with a broader spectrum of non-AIDS-defining medical and surgical illnesses. Hospital mortality was observed in 28 patients (8.6%), of whom 17 (61.0%) had CD4 lymphocyte counts below 200 cells/µL, and 20 (71.4%) were admitted with ADIs. Tuberculosis was the most frequent opportunistic infection among patients who died during hospitalizations.

**Table 3:** Description of clinical syndromes (N = 325) ^*^Includes Cryptococcal meningitis, encephalopathy and Toxoplasmosis; ^**^Includes chronic diarrhea and esophageal candidiasis; OI: opportunistic infection

Clinical syndrome	Under 200 cells/µL (N = 1141), n (%)	Over 200 cells/µL (N = 1781), n (%)	Unknown CD4 count (N = 331), n (%)	Total
AIDS-defining illnesses				
Tuberculosis				
Pulmonary	19 (16.7%)	19 (10.7%)	4 (12.1%)	42
Extrapulmonary	26 (22.8%)	23 (12.9%)	3 (9.1%)	52
Disseminated	18 (15.8%)	8 (4.5%)	2 (6.1%)	28
Central Nervous System OIs *	12 (10.5%)	3 (1.7%)	0	15
Gastrointestinal OIs **	8 (7.0%)	4 (2.2%)	0	12
Pneumocystis jirovecii pneumonia	5 (4.3%)	3 (1.7%)	1 (3.0%)	9
Cytomegalovirus retinitis	5 (4.3%)	3 (1.7%)	0	8
Miscellaneous	0	2 (1.1%)	2 (6.1%)	1
Non-AIDS-defining illnesses				
Medical illness				
Cardiovascular disorders	7 (6.1%)	11 (6.2%)	3 (9.1%)	21
Liver disorders	4 (3.5%)	8 (4.5%)	1 (3.0%)	13
Gastrointestinal disorders	3 (2.6%)	8 (4.5%)	1 (3.0%)	12
Renal disorders	1(0.9%)	11 (6.2%)	1 (3.0%)	13
Miscellaneous	10 (8.8%)	27 (15.2%)	5 (15.2%)	42
Surgical illness	9 (7.9%)	38 (21.3%)	6 (18.2%)	53
Pregnancy or childbirth	1 (0.9%)	10 (5.6%)	2 (6.1%)	13
Accidents	3 (2.6%)	6 (3.4%)	1 (3.0%)	10

## Discussion

Our study provides an insight into the evolving profile of PLHIV undergoing hospitalization in the ART era, highlighting the persistent burden of advanced HIV disease and ADIs, despite widespread ART coverage.

The study demonstrates a predominance of middle-aged adults, with a median age of 44 years (IQR = 19), reflecting the aging of the HIV population in the ART era. Similar patterns have been reported across other developing countries, where hospitalized PLHIV remain comparatively younger than in developed countries but show a clear shift towards middle age [16]. Owachi et al. [19] reported a median age of 39 years among hospitalized PLHIV in Uganda, while Liu et al. [5] observed an increase in the median age from 31 years among ART-naïve individuals to 39 years among those on ART for more than six months in China, with nearly one-fifth of hospitalizations occurring in patients aged 50 years or older. In Cameroon, Fodop et al. [20] documented a mean age of 44 years, with approximately half of hospitalizations occurring among individuals aged 40-59 years [10]. Similarly, in India, Kumar et al. [21] have projected a steady increase in the mean age of PLHIV from 38.4 years in 2005 to 45.5 years by 2025, driven by expanded ART coverage and improved survival. In contrast, developed countries report the median age among hospitalized PLHIV to be substantially higher, indicating a clear epidemiological transition. In London, Rein et al. [8] reported a median age of 52 years, while in Italy, Pipito et al. [7] documented a mean age of 46.3 years (SD = 13) among hospitalized PLHIV. As PLHIV age, they experience slower immunological recovery, alongside an increasing prevalence of NCDs, often compounded by polypharmacy and heightened risk of drug-drug interactions. Collectively, these findings indicate a transition in HIV-related hospitalization, with younger individuals more likely to present with ADIs and older PLHIV increasingly hospitalized for non-AIDS-defining and non-communicable conditions, highlighting the need for integrated care models that address chronic disease management, geriatric considerations, and long-term ART-related complications alongside traditional opportunistic infection control.

Our study showed a slight male predominance, especially in treatment-naive patients (92, 66.7%) and ADI hospitalizations (102, 69.4%). This is consistent with findings from several international cohorts and may reflect delayed health-seeking behaviour, late HIV diagnosis, or poorer engagement with HIV care among men [4,22]. Rein et al. [8] observed a male majority in London, where 64% of hospitalizations were among men who have sex with men, while Burke et al. [3] reported that nearly two-thirds (68.7%) of hospitalized PLHIV globally were males [6]. Similarly, Liu et al. [5] documented a striking male predominance (91.7%) among hospitalized PLHIV in China, with consistently high proportions across ART-naïve and ART-experienced groups, and female sex was associated with a significantly lower risk of AIDS-defining event-related hospitalization. In Italy, Pipito et al. [7] reported that 71.6% of hospitalizations occurred among males, attributing sex differences partly to regional transmission dynamics, with homosexual transmission predominating in developed countries and contributing to higher male hospitalization rates [6]. In contrast, studies from sub-Saharan Africa have reported female predominance among hospitalized PLHIV. Owachi et al. [19] found that 56.5% of admissions in Uganda involved women, while Fodop et al. [20] reported a similar female predominance (57.8%) in Cameroon. These differences likely reflect contextual factors, including higher HIV prevalence among women in these countries.

The overall median CD4 lymphocyte count was 286.5 cells/µL (IQR = 416), which is higher than that observed in other developing countries, such as 109 cells/µL, as reported by Owachi et al. [19] in Uganda, while global meta-analysis by Burke et al. [3] demonstrated a weighted median CD4 lymphocyte count of 111 cells/µL across diverse regions. However, marked differences in immunological status were observed between treatment-naïve and ART-experienced patients in the present study. More than half of treatment-naïve patients (54%) presented with CD4 lymphocyte counts below 200 cells/µL, compared with 19% of those receiving ART. Liu et al. [5] reported a similar pattern in China, with a lower median CD4 lymphocyte count of 108 cells/µL at admission, but more than half of patients had CD4 lymphocyte counts below 200 cells/µL, particularly those who were ART naïve and those early in treatment. In contrast, Rein et al. [8] documented a median CD4 lymphocyte count of 510 cells/µL in a London cohort, reflecting earlier diagnosis and sustained treatment engagement.

Overall, 138 (42.5%) of PLHIV in our study were treatment naïve at the time of hospitalization. We also observed that patients admitted with ADIs were more likely to have been on ART for less than six months, whereas those on ART for more than two years were predominantly admitted for non-ADI causes. Similar patterns have been reported across diverse settings. Owachi et al. [19] reported that 36% of patients had interrupted ART for more than three months or were treatment naïve at the time of hospitalization. Such patients were at a higher risk of mortality. Burke et al. [3] reported approximately 60% of hospitalized PLHIV to be receiving ART at hospitalization. Liu et al. [5] reported a substantially higher proportion of treatment-naïve patients (70%), with such people having a longer length of stay in the hospital and higher health care costs. In contrast, Rein et al. [8] in London reported that only 4.7% of patients were not on ART, suggesting a difference in patterns between developed and developing countries.

In the present study, ADIs were observed in nearly half (147, 45.2%) of all hospitalizations, with tuberculosis (including pulmonary, extrapulmonary, and disseminated tuberculosis) emerging as the predominant cause (53, 36.3% of all ADIs). This highlights the continued burden of tuberculosis among PLHIV in our setting, particularly among patients with CD4 lymphocyte counts below 200 cells/µL. Opportunistic infections involving the central nervous system and gastrointestinal tract were also largely confined to patients with lower CD4 counts, reinforcing the strong link between immunological status and HIV-related morbidity [23]. Similar findings were reported by Ford et al. [6], with ADIs accounting for nearly half of adult hospitalizations, alongside a substantial burden of severe bacterial infections. Liu et al. [5] reported 60% of hospitalizations due to AIDS-defining events, predominantly Pneumocystis pneumonia and tuberculosis, and with a high risk among those with advanced HIV infection. While non-AIDS-defining conditions, including cardiovascular, hepatic, renal, gastrointestinal, surgical, obstetric, and trauma-related hospitalizations, constituted the other half of hospitalizations, ADIs still contributed a substantial proportion, in contrast to developed countries, where non-AIDS conditions now predominate. The London cohort described by Rein et al. [8] demonstrates a marked epidemiological transition, with only a small proportion of hospitalizations attributable to ADIs and the majority due to NCDs and degenerative conditions. Similar shifts towards non-AIDS-related hospitalizations have been reported from Thailand [15] and Italy [7], where older PLHIV are increasingly admitted for cardiovascular, hepatic, renal, and malignant conditions.

Hospital mortality in the present study was seen in 28 patients (8.6%), with deaths occurring predominantly among patients with advanced HIV disease and ADIs, with tuberculosis emerging as the most frequent opportunistic infection among patients who died. Compared with other studies from developing countries, the mortality observed in this cohort was lower than that reported by Owachi et al. [19] in Uganda (26%) and Fodop et al. [20] in Cameroon (26.1%), as well as the pooled adult mortality of 20% reported by Ford et al. [6], and the global estimate of 16.2% described by Burke et al. [3]. These higher mortality rates in other settings have been attributed to delayed presentation, severe immunosuppression, and limited access to critical care services, with deaths commonly resulting from renal insufficiency, central nervous system infections, tuberculosis, diarrheal diseases, and pneumonia [4,24,25]. In contrast, mortality rates reported from high-income settings are substantially lower; Pipito et al. [7] documented a hospital mortality of 6%, with deaths primarily related to wasting syndromes, neoplasms, and cardio-respiratory conditions rather than acute opportunistic infections. The comparatively lower mortality observed in the present study may reflect improved inpatient management, wider ART availability, and earlier presentation among a subset of patients; however, the continued concentration of deaths among individuals with low CD4 lymphocyte counts and ADIs highlights persistent gaps in HIV diagnosis and sustained treatment engagement.

Limitations

Our study had certain limitations. It was conducted among hospitalized PLHIV and, therefore, represents a sicker subset of the HIV population. The burden of advanced HIV disease may be overestimated. The cross-sectional study design limits causal inference. The documentation of CD4 accounts was incomplete, with missing values for 33 patients. Post-discharge outcomes and long-term survival were not assessed.

## Conclusions

Despite high ART coverage, ADIs, particularly tuberculosis, continue to contribute substantially to hospitalization and hospital mortality among PLHIV. Concurrently, the rising burden of non-ADIs and NCDs among an aging PLHIV population highlights the need for integrated, age-responsive HIV care models.
